# Comparative Digestive Behavior, Chemical Profiles, and Antioxidant Activities of Coicis Semen Ultrafine Powder Suspension and Water Decoction During In Vitro Gastrointestinal Digestion

**DOI:** 10.3390/foods15203711

**Published:** 2026-10-19

**Authors:** Yadi Hou, Jun Chi, Haiyang Du, Yixiao Li, Qi Wang, Bingbing An, Manliang Chen, Liping Dai

**Affiliations:** 1 Henan Collaborative Innovation Center for Research and Development on the Whole Industry Chain of Yu-Yao, Henan University of Chinese Medicine, Zhengzhou 450046, China; 2 Engineering Technology Research Center for Comprehensive Development and Utilization of Authentic Medicinal Materials, Zhengzhou 450046, China

**Keywords:** Coicis Semen, ultrafine powder suspension, INFOGEST 2.0, lipid metabolism, antioxidant activity, metabolomics

## Abstract

This study compared the digestive behavior, chemical profiles, and antioxidant activity of a Coicis Semen ultrafine powder suspension (CSUP) and water decoction (CSW) during in vitro gastrointestinal digestion using the INFOGEST 2.0 model. Peptide content, lipid profile changes, metabolite profiles, and antioxidant capacity were analyzed by peptide quantification, ultra-performance liquid chromatography–tandem mass spectrometry (UPLC-MS/MS)-based lipidomics, untargeted metabolomics, and 2,2-diphenyl-1-picrylhydrazyl (DPPH) and 2,2′-azino-bis(3-ethylbenzothiazoline-6-sulfonic acid) (ABTS) radical-scavenging assays. Chemical similarity and antioxidant-related differences were further evaluated using study-specific exploratory indices, including the Chemical Equivalence Index (CE*), Bioequivalence Index (BE), and Chemical–Biological Deviation Index (CBDI*). Compared with CSW, CSUP exhibited approximately 4–8 times higher measured peptide-related content during gastric digestion and 7–10 times higher measured peptide-related content during intestinal digestion. Lipidomics revealed stage-dependent changes in the relative MS signal proportions of triacylglycerols (TAGs), diacylglycerols (DAGs), and other lipid-related components, with pronounced changes occurring during the intestinal phase. Metabolomics revealed different profiles between CSUP and CSW, with differential metabolites mainly enriched in steroid metabolism and cytochrome P450-related pathways according to pathway enrichment analysis. CSUP showed lower DPPH and ABTS IC_50_ values and higher BE values, consistent with greater in vitro radical-scavenging activity under the tested conditions, while CBDI* values increased during the early intestinal phase. Overall, CSUP and CSW exhibited different digestion-associated characteristics under the tested conditions, and CSUP showed higher measured peptide-related content and stronger in vitro radical-scavenging activity than CSW. These findings provide comparative information for evaluating different preparation forms of Coicis Semen.

## 1. Introduction

Coicis Semen is the dried mature seed kernel of *Coix lacryma-jobi* L. var. ma-yuen (Roman.) Stapf and has long been used as both medicinal and edible resource. Modern studies have shown that Coicis Semen contains starch, proteins, fatty oils, polysaccharides, sterols, phenolic acids, and other bioactive small molecules, and exhibits antioxidant, anti-inflammatory, immunomodulatory, and lipid metabolism-regulating activities [1,2,3,4]. Among these constituents, fatty oils and glycerides are characteristic components of Coicis Semen, while peptides released from protein digestion may also contribute to its antioxidant potential [5,6]. However, the functional properties of food-medicine materials depend not only on their initial chemical composition, but also on their release, degradation, transformation, and bioaccessibility during gastrointestinal digestion.

In plant seed matrices, proteins, lipids, and starch are often embedded within cell walls or starch–protein–lipid complexes, thereby limiting enzyme accessibility. Food structure and processing can therefore markedly affect nutrient release and digestion kinetics; materials with similar chemical compositions may produce different digestive responses when their physical structures differ [7,8]. Ultrafine grinding is a physical modification technique that reduces particle size, increases specific surface area, disrupts cell wall structures, and improves powder dispersibility, thereby affecting dissolution, digestion, and functional properties [9,10,11]. For Coicis Semen, which is rich in starch, proteins, and lipids, ultrafine grinding may influence protein hydrolysis, measured peptide-related content, lipid transformation, and the release of lipophilic bioactive compounds.

The INFOGEST static in vitro digestion model provides standardized digestive fluid composition, enzyme activity, pH, and operating procedures, and is widely used to evaluate food digestion, nutrient release, and bioaccessibility [12,13]. Although it cannot fully reproduce the dynamic processes of gastric emptying, intestinal absorption, or microbial fermentation in vivo, its high reproducibility makes it suitable for comparing the digestive behavior of different food structures or processing forms. During lipid digestion, bile salts, pancreatic lipase/colipase, and the oil–water interface jointly regulate the hydrolysis of triacylglycerols (TAGs) into monoacylglycerols (MAGs) and free fatty acids (FFAs) [14,15]. Therefore, combining the INFOGEST model with lipidomics, metabolomics, and antioxidant activity assays can provide a systematic understanding of the digestive and metabolic characteristics of Coicis Semen ultrafine powder (CSUP).

In addition, gastrointestinal digestion of dietary proteins releases small peptides with antioxidant potential, and their antioxidant capacity is associated with amino acid composition, peptide sequence, molecular weight, and hydrophobicity [6]. Although DPPH and ABTS assays are chemical antioxidant assays and cannot be directly equated with in vivo antioxidant effects, they provide preliminary characterization of the in vitro reducing and hydrogen-donating capacity of digestion products [16,17]. Therefore, integrating measured peptide-related content, lipid transformation, metabolomic profiling, and antioxidant indicators may better reflect the effects of processing on functional food quality than single-component or single-activity evaluation.

Against this background, the present study compared two preparation forms of Coicis Semen: a water decoction (CSW) and ultrafine powder suspension (CSUP). These preparations were derived from the same starting material but differed in processing and matrix state. Using the INFOGEST 2.0 in vitro gastrointestinal digestion model, we compared their peptide-related content, lipid profiles, metabolite profiles, and in vitro antioxidant activity during digestion. The aim was to characterize differences between the two preparation forms rather than to isolate the independent effect of ultrafine grinding. These findings may provide experimental evidence for the quality evaluation of ultrafine Coicis Semen products and the development of functional foods.

## 2. Materials and Methods

### 2.1. Source of Samples and Reagents

Coicis Semen was provided by Beijing Tongrentang (Group) Co., Ltd. (Beijing, China). The BeyoBCA Peptide Concentration Assay Kit was purchased from Shanghai Biyuntian Biotechnology Co., Ltd. (Shanghai, China). Pepsin (1:10,000, activity ≥ 3000 U/g) and pancreatic enzymes (activity ≥ 250 U/mg) were purchased from Shanghai Macklin Biochemical Co., Ltd. (Shanghai, China). Triacylglycerol reference standard, vitamin C, porcine bile powder, DPPH reagent, and ABTS reagent were purchased from Shanghai Yuanye Bio-Technology Co., Ltd. (Shanghai, China). Potassium persulfate was purchased from Shanghai Yien Chemical Technology Co., Ltd. (Shanghai, China). Methanol, acetonitrile, and isopropanol were purchased from Thermo Fisher Scientific (Waltham, MA, USA). Dichloromethane was purchased from Tianjin Zhiyuan Chemical Reagents Co., Ltd. (Tianjin, China). Ultrapure water was supplied by Hangzhou Wahaha Group Co., Ltd. (Hangzhou, China).

### 2.2. Preparation of Coicis Semen Ultrafine Powder

After impurities were removed, the Coicis Semen raw material was dried at 60 °C to constant weight. The dried material was processed using an LSF-3 continuous classifier mill (Shanghai Tianhe, Shanghai, China) at 5200 r/min under water cooling to limit the temperature increase during grinding. The ground material was subsequently sieved to obtain an ultrafine powder with a particle size of less than 50 μm. The resulting powder was sealed, protected from light, and stored in a desiccator until use.

### 2.3. Preparation of Samples for In Vitro Digestion

Two preparations were used: CSW and a suspension of CSUP prepared as described in Section 2.2. Both were standardized to the same starting-material equivalent (0.10 g/mL), which does not imply equal protein, lipid, or total solid inputs into digestion. For CSW, Coicis Semen (100 g) was decocted in an earthenware pot with 800 mL of water under gentle boiling conditions for 30 min, timed from the onset of boiling, without a reflux condenser. The decoction was filtered through a nonwoven fabric filter bag, and the residue was re-extracted with 600 mL of water under the same conditions. The filtrates were combined and diluted to 1000 mL. For the CSUP suspension, CSUP (100 g) was dispersed in water and diluted to 1000 mL. The suspension was thoroughly mixed before sampling to minimize variation caused by particle sedimentation.

### 2.4. INFOGEST 2.0 In Vitro Simulation of Gastrointestinal Digestion

Gastrointestinal digestion was performed according to the standardized INFOGEST 2.0 protocol [12], with the oral phase omitted because the present study focused on gastric and intestinal digestion. Simulated gastric fluid (SGF) and simulated intestinal fluid (SIF) were prepared according to the INFOGEST 2.0 protocol and adjusted to pH 3.0 and 7.0, respectively. Detailed compositions and preparation procedures for SGF and SIF are provided in Table A1 and Table A2 of the Appendix A, respectively.

#### 2.4.1. Sequential Simulated Gastric and Intestinal Digestion

An aliquot of the CSW or CSUP sample was mixed with prewarmed SGF at a volume ratio of 1:1. Pepsin was added to achieve a final activity of 2000 U/mL in the digestion mixture. The mixture was incubated at 37 °C with continuous shaking at 120 rpm. Samples were collected at 0, 30, 60, 90, and 120 min, with the 0-min sample collected immediately after SGF addition and pH adjustment.

After gastric digestion, the remaining gastric digest was mixed with prewarmed SIF at a volume ratio of 1:1. Bile salts were added to a final concentration of 10 mM, and trypsin and lipase were added to final activities of 100 and 2000 U/mL, respectively. The mixture was incubated at 37 °C with continuous shaking at 120 rpm. Samples were collected at 0, 30, 60, 120, and 180 min. The intestinal 0 min sample was collected immediately after the addition of SIF, bile salts, and digestive enzymes and completion of pH adjustment, before the timed intestinal incubation began.

#### 2.4.2. Sampling and Digestion Termination

After sampling at each predetermined digestion time point, the samples were immediately placed in a boiling water bath to inactivate enzymes and terminate gastrointestinal digestion. The inactivated samples were centrifuged at 4 °C, and the supernatant was collected and freeze-dried. The resulting freeze-dried samples were used as standardized materials for subsequent assays, including peptide content determination, lipid-related component analysis, untargeted metabolomics, and DPPH and ABTS antioxidant activity assays.

### 2.5. Determination of Peptide Content During Digestion

The assay was performed according to the instructions of the peptide concentration assay kit. A calibration curve was constructed using the standard provided with the kit over the concentration range of 0–1000 μg/mL. The linear regression equation was *Y* = 0.0021 *X* + 0.056 (*R*^2^ = 0.9983), where *X* represents the standard concentration (μg/mL) and *Y* represents the absorbance. The absorbance of samples collected at different digestion stages was then measured. Background signals from the enzyme and digestive fluid blanks were subtracted from the sample values. Peptide content was calculated based on the dry weight of the starting Coicis Semen material and expressed as mg/g.

### 2.6. UPLC-MS/MS Analysis of Lipid Components

#### 2.6.1. Preparation of Sample Solutions

Freeze-dried samples (0.6 g) were accurately weighed, placed in a stoppered conical flask, and mixed with 50 mL of acetonitrile–dichloromethane (65:35, *v*/*v*). The flask was weighed, and the sample was soaked for 2 h before ultrasonic extraction for 30 min (300 W, 50 kHz). After cooling, the stoppered flask and its contents were reweighed, and acetonitrile–dichloromethane (65:35, *v*/*v*) was added to restore the total mass recorded before extraction. The extract was then mixed thoroughly and filtered. The resulting filtrate was centrifuged at 4 °C and 8000 r/min for 10 min, and the supernatant was collected for UPLC-MS/MS analysis.

#### 2.6.2. Chromatographic Conditions

A Thermo Synchronis C18 column (100 × 2.1 mm, 1.7 μm) was used with acetonitrile–isopropanol (1:1, *v*/*v*) as the mobile phase under isocratic elution at a flow rate of 0.3 mL/min, a column temperature of 30 °C, and an injection volume of 2 μL.

#### 2.6.3. Mass Spectrometry Conditions

An Orbitrap Exploris 120 mass spectrometer (Thermo Fisher Scientific, USA) equipped with an atmospheric pressure chemical ionization (APCI) source was operated in positive ion mode. The mass spectrometer was operated at a resolution of 60,000 with a scan range of *m*/*z* 100–1500 and a mass tolerance of <5 ppm. The ion source temperature was set at 350 °C, the ion transfer tube temperature was 300 °C, and the positive ion current was 4 μA. The sheath gas, auxiliary gas, and purge gas flow rates were set at 35, 5, and 1 Arb, respectively. Data-dependent MS/MS acquisition (dd-MS^2^) was employed for metabolite fragmentation. Raw data were processed using Compound Discoverer 3.2 for peak extraction, peak alignment, noise reduction, and peak area integration. Metabolite annotation was performed based on accurate mass, MS/MS fragment ions, and database matching against the Human Metabolome Database (HMDB) and METLIN (https://hmdb.ca/, http://metlin.scripps.edu). Metabolites confirmed by authentic standards were considered definitively identified, whereas compounds annotated only by database matching were reported as tentative annotations.

### 2.7. Untargeted Metabolomics Analysis During Digestion

Three replicate samples (*n* = 3) were collected at each digestion stage for untargeted metabolomics analysis using the same UPLC-MS/MS conditions described in Section 2.6. Raw data were processed using Compound Discoverer 3.2 for peak extraction, alignment, noise reduction, and peak-area integration. Principal component analysis (PCA) and partial least squares discriminant analysis (PLS-DA) were performed, and the PLS-DA model was evaluated by leave-one-out cross-validation and a 100-permutation test. Differential metabolites were screened using VIP > 1 and *p* < 0.05. Metabolite annotation was based on exact mass, MS/MS fragmentation, Human Metabolome Database (HMDB), and METLIN. Enrichment analysis was performed using MetaboAnalyst with reference to HMDB and KEGG, with *p* < 0.01 considered significant.

### 2.8. Evaluation of Antioxidant Activity

#### 2.8.1. Determination of DPPH Radical Scavenging Activity

Briefly, the samples were diluted with water to different concentrations within the range of 2–60 μg/mL. Then, 1 mL of sample solution at different concentrations was mixed with 1 mL of 0.1 mmol/L DPPH ethanol solution, vortexed thoroughly, incubated in the dark for 30 min, and measured at 519 nm. Vitamin C was used as the positive control to calculate the DPPH radical scavenging rate. The scavenging rate (%) was calculated as follows: (1 − A sample/A blank) × 100%. Each concentration was measured in triplicate, and the half-maximal inhibitory concentration (IC_50_) was calculated.

#### 2.8.2. Determination of ABTS Radical Scavenging Activity

ABTS working solution was prepared in advance by mixing 7.4 mmol/L ABTS solution with 2.6 mmol/L potassium persulfate solution at a volume ratio of 1:1, followed by incubation in the dark for 12 h. The mixture was diluted with absolute ethanol to an absorbance of 0.70 ± 0.02 at 734 nm. The samples were diluted with water to different concentrations within the range of 2–60 μg/mL. Sample solution (1 mL) at different concentrations was mixed with 3 mL of ABTS working solution and vortexed thoroughly. After incubation in the dark for 30 min, absorbance was measured at 734 nm. Vitamin C was used as the positive control to calculate the ABTS radical scavenging rate and IC_50_ value. Each concentration was measured in triplicate.

### 2.9. Evaluation of Chemical Equivalence, Bioequivalence and Comprehensive Index

To comprehensively evaluate differences in chemical composition and biological effects between CSUP and CSW during in vitro digestion, three study-specific exploratory indices were established: the Chemical Equivalence Index (CE*), Bioequivalence Index (BE), and Chemical–Biological Deviation Index (CBDI*). The detailed calculation formulas are described in Table A3.

CE* was used to assess the similarity in chemical composition between the two sample groups at identical digestion stages. The included indicators comprised measured peptide-related content, lipid classes (relative abundances of TAGs, DAGs, and other lipids), and relative peak areas of representative differential metabolites. Because these indicators were expressed in different units or analytical scales, all indicators were normalized to dimensionless values before calculation and were not directly summed in their original units. CE* was calculated according to Equation (1):

(1) $$CE^{*}=1-\sum_{i=1}^{n}w_{i}\cdot \left|\frac{C_{i}^{S}-C_{i}^{R}}{C_{i}^{S}+C_{i}^{R}+\varepsilon }\right|$$

where ($C_{i}^{S}$) and ($C_{i}^{R}$) denote the relative content or peak area of the (*i*)-th constituent in the CSUP and CSW groups, respectively; (*w_i_*) is its weight, (*n*) is the number of constituents, and (*ε*) is a small constant. A CE* value closer to 1 indicates higher chemical similarity. Peptides, lipids, and metabolites were assigned equal category weights, with equal weighting applied to individual indicators within each category.

BE was used to evaluate the composite in vitro radical-scavenging activity of the two sample groups. The included indicators were DPPH and ABTS radical scavenging capacities. Because a lower IC_50_ value corresponds to stronger antioxidant activity, BE was calculated according to Equation (2):

(2) $$\mathrm{BE}=\sum_{j=1}^{m}v_{j}\cdot \frac{B_{j}^{S}}{B_{j}^{R}}$$

where ($B_{j}^{S}$) and ($B_{j}^{R}$) denote the (j)-th radical-scavenging indicator in the CSUP and CSW groups, (*v_j_*) is its weight, and (*m*) is the number of indicators. DPPH and ABTS were equally weighted (*v_j_* = 0.5). A BE value > 1 indicates greater composite in vitro radical-scavenging activity in CSUP relative to CSW. BE is a study-specific exploratory index and does not represent conventional bioequivalence or general biological activity.

CBDI* was used to evaluate the degree of correspondence between changes in chemical composition and changes in the composite in vitro radical-scavenging activity, and was calculated as follows:

(3) $$\mathrm{CBD}I^{*}=\frac{\mathrm{BE}}{CE^{*}+\varepsilon }$$

A higher CBDI* value may result from a lower CE*, a higher BE, or both, whereas a lower value indicates a closer numerical relationship between the two indices. CBDI* was used only for descriptive comparison across digestion stages and does not indicate a causal chemical–biological relationship.

### 2.10. Statistical Analyses

All experimental data were statistically analyzed using SPSS 26.0 and expressed as mean ± standard deviation ($\bar{x}\;\bar{x}$ ± s). One-way analysis of variance (ANOVA) was used for comparisons among multiple groups, and *p* < 0.05 was considered statistically significant. Release curves, antioxidant activity curves, heatmaps, PCA/PLS-DA plots, and correlation network diagrams were generated using OriginPro 2026, GraphPad Prism 8.2.1, or R software 4.6.1.

## 3. Results

### 3.1. Dynamic Changes in Peptide Content During In Vitro Gastrointestinal Digestion

**Figure 1:**
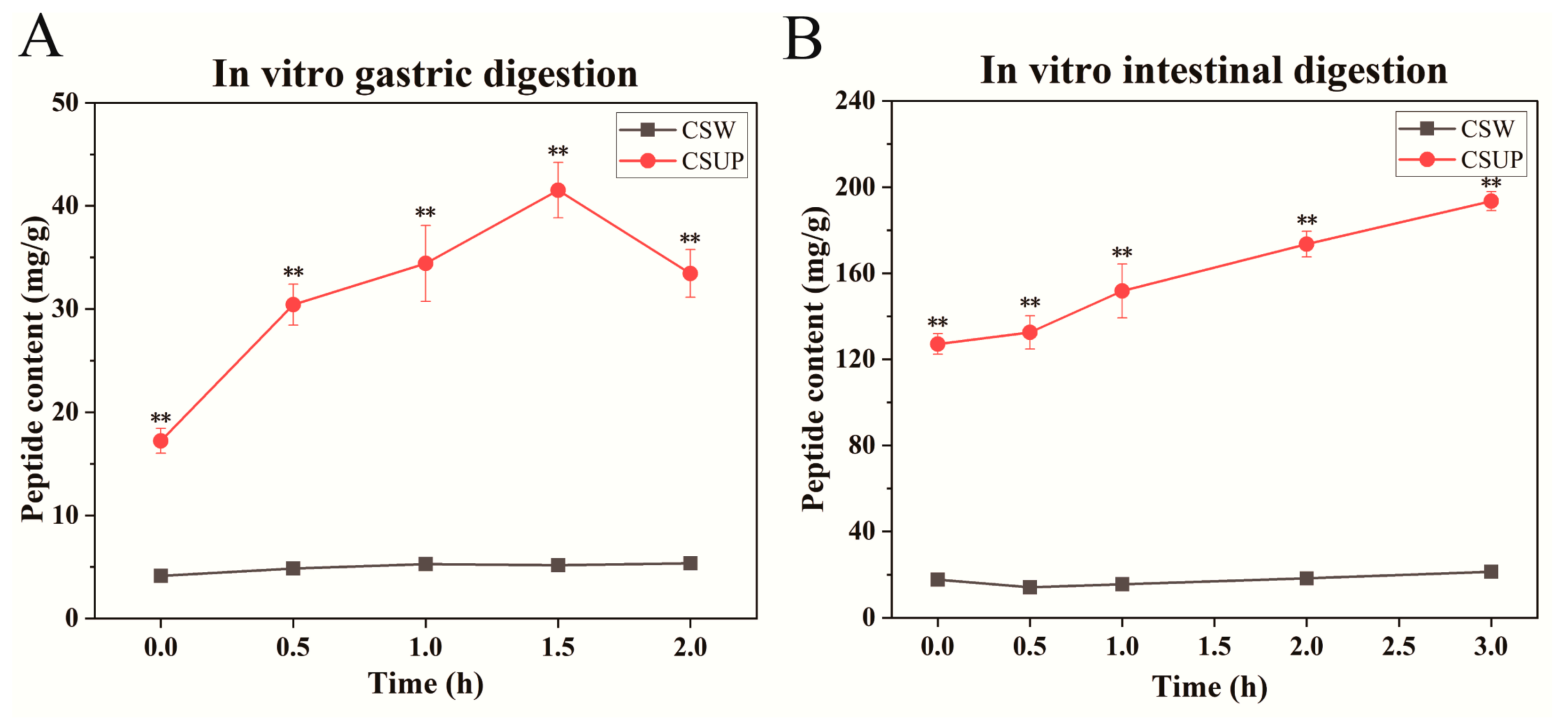
Changes in peptide content during simulated gastrointestinal digestion. (**A**) Simulated gastric digestion; (**B**) Simulated intestinal digestion. Gray squares and lines represent CSW, while red circles and lines represent CSUP. Data are presented as mean ± SD (*n* = 3), with error bars indicating SD. ** *p* < 0.01 vs. CSW at the same time point.

During simulated gastrointestinal digestion, CSUP showed higher measured peptide content than CSW at most time points (Figure 1). During gastric digestion, peptide content in CSUP increased to a maximum at 1.5 h and then slightly decreased at 2 h, while CSW remained relatively low. CSUP values were approximately 4–8 times those of CSW during this phase. At intestinal 0 min, both groups showed an abrupt increase relative to gastric 2 h. Because this sample was collected after addition of SIF, bile salts, digestive enzymes, and pH adjustment, this discontinuity was not interpreted as immediate measured peptide-related content. During subsequent intestinal digestion, CSUP remained higher than CSW, with values approximately 7–10 times higher by the end of digestion.

### 3.2. Variations in Lipid Components During In Vitro Digestion

**Figure 2:**
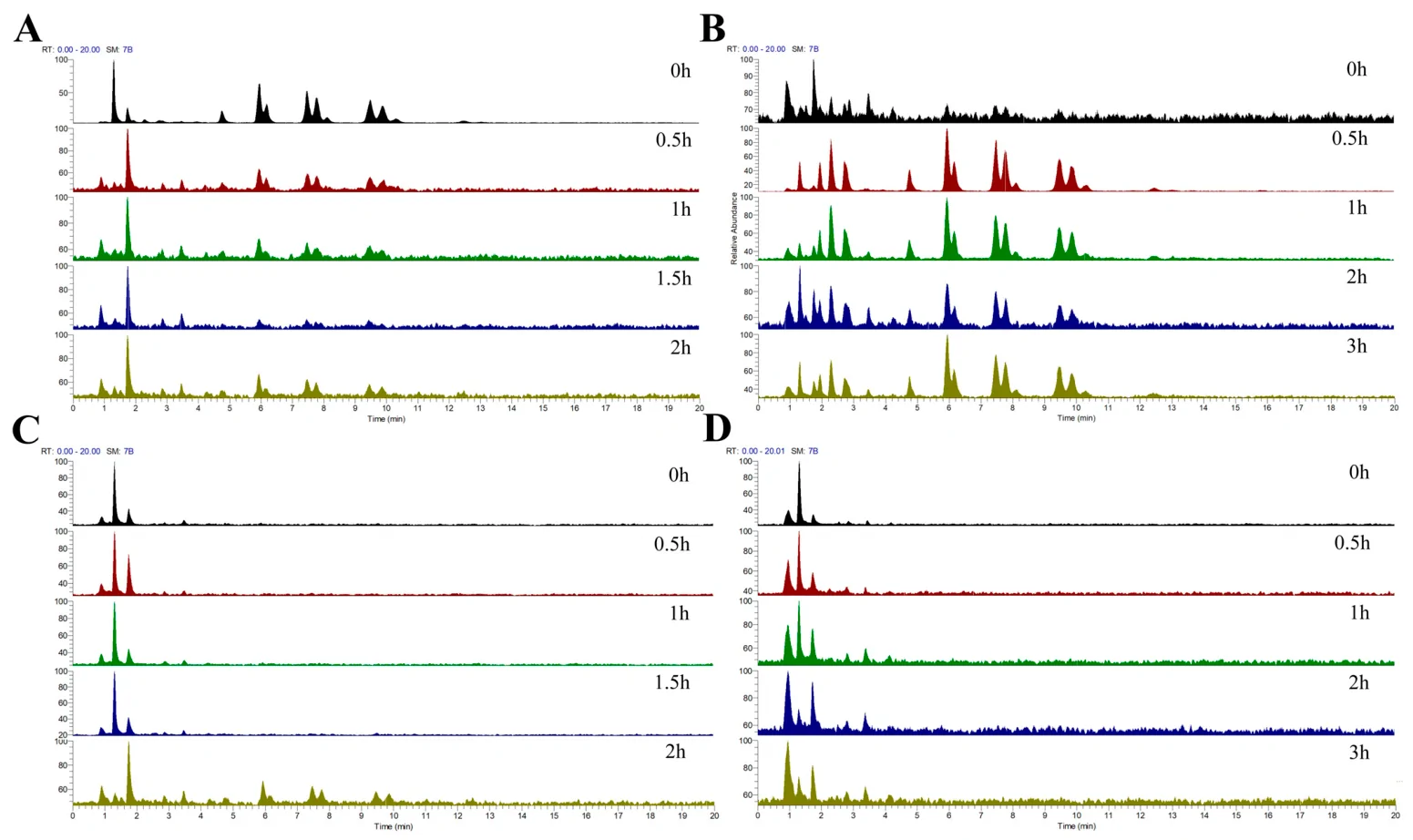
Total ion chromatograms of CSUP and CSW at each stage of simulated in vitro digestion. (**A**) Simulated gastric digestion of CSUP; (**B**) Simulated intestinal digestion of CSUP; (**C**) Simulated gastric digestion of CSW; (**D**) Simulated intestinal digestion of CSW.

To compare lipid transformation characteristics among different preparation forms during in vitro digestion, lipid-related components in the digestion samples were analyzed by UPLC-MS/MS. A total of 43 lipid-related features, mainly comprising TAGs, DAGs, and other lipid components, were detected (Table 1). Only compounds confirmed using authentic standards were considered definitively identified. Representative total ion chromatograms (TICs) showed that lipid signals in all samples were mainly concentrated within 0–10 min, and differences in peak distribution and relative response intensity were observed between CSUP and CSW at different digestion stages (Figure 2).

**Figure 3:**
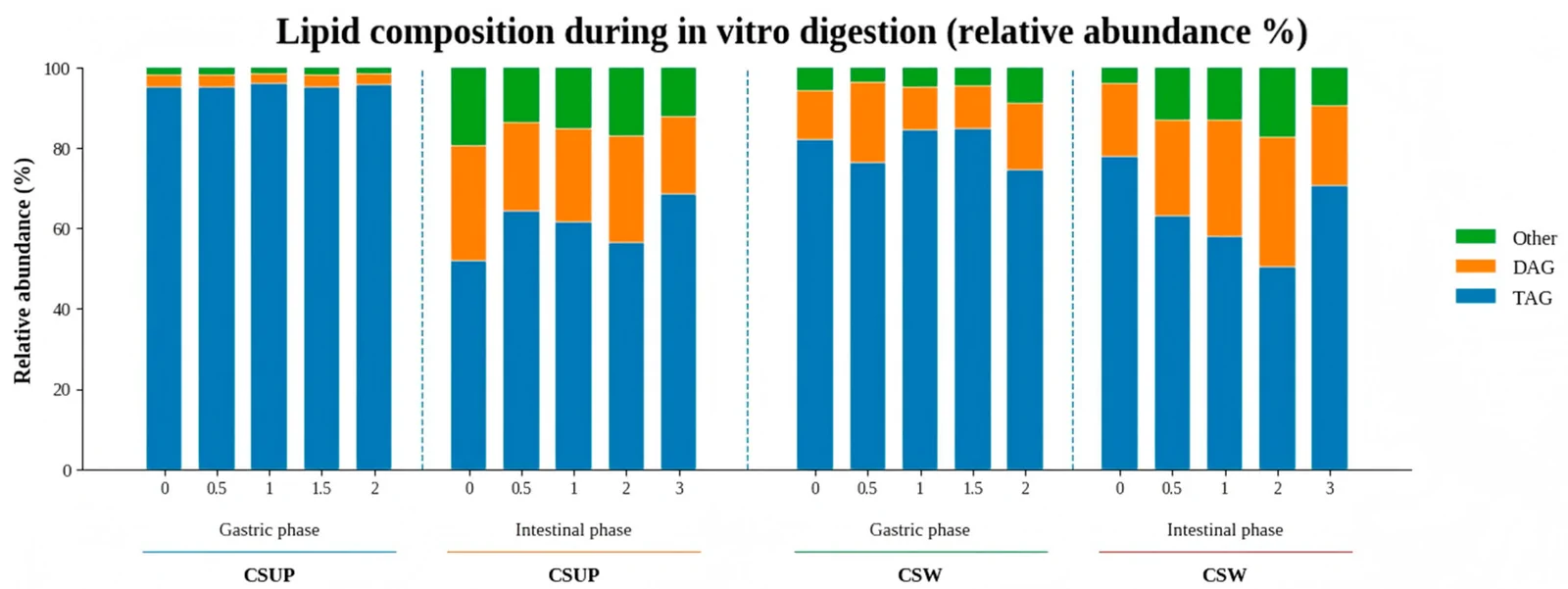
Changes in lipid classes during in vitro digestion.

Further analysis of the relative lipid-class signal proportions revealed that TAGs were predominant in CSUP during gastric digestion and remained consistently high, whereas DAGs and other lipid-related signals remained relatively low. CSW was also dominated by TAG-related signals during gastric digestion but showed different relative distributions of DAGs and other lipid-related components compared with CSUP. During intestinal digestion, the relative TAG signal proportion in CSUP increased rather than decreased, while the relative distributions of DAGs and other lipid-related components also changed with digestion time. CSW showed a different stage-dependent pattern of relative lipid-class signals during intestinal digestion (Figure 3).

Overall, CSUP and CSW exhibited different stage-dependent patterns in the relative MS signal distribution of lipid classes during in vitro digestion, with pronounced changes occurring during the intestinal phase.

### 3.3. Characteristics of Differential Metabolites Revealed by Untargeted Metabolomics

**Figure 4:**
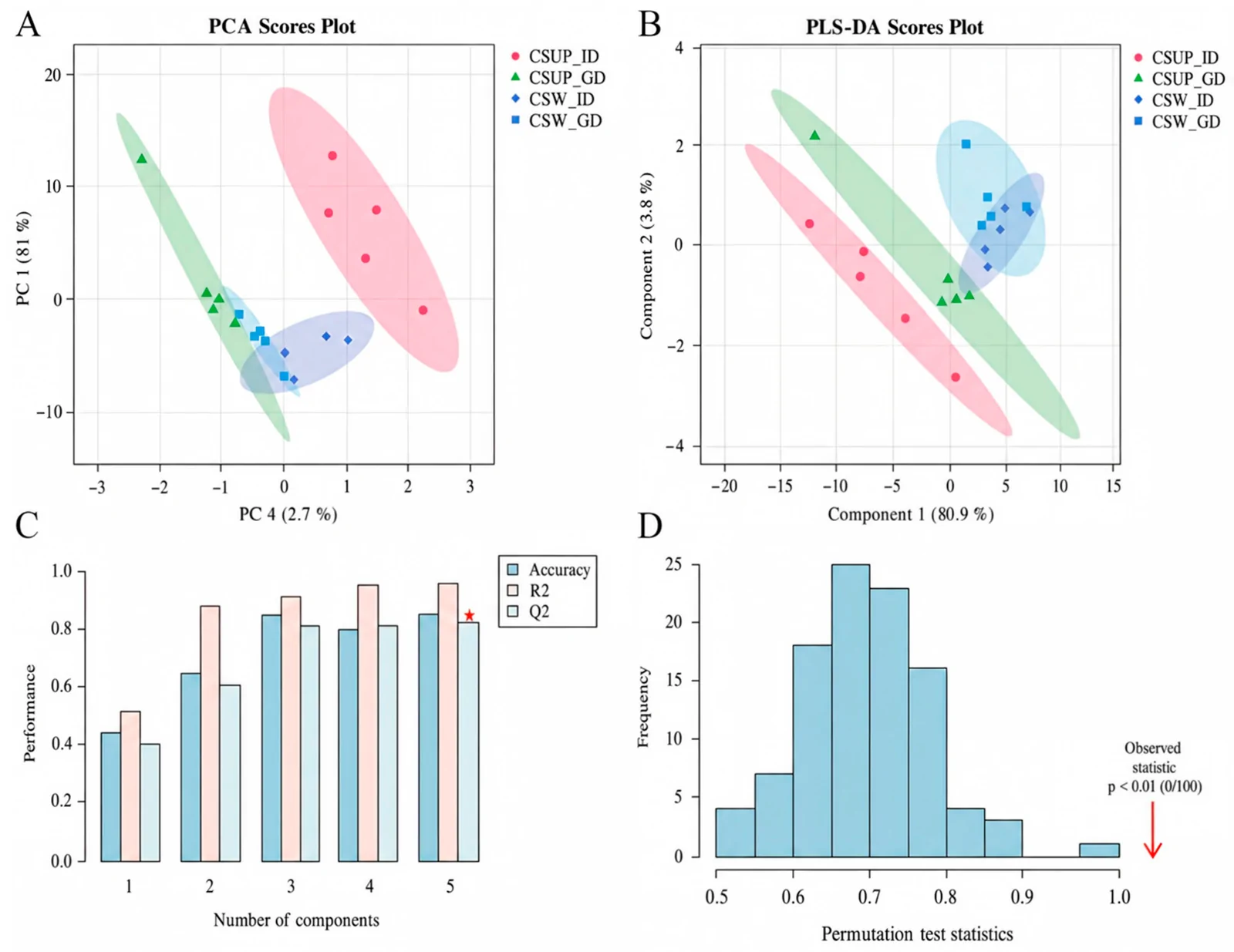
PCA and PLS-DA multivariate statistical analyses of samples from each digestion stage. (**A**) PCA score plot; (**B**) PLS-DA score plot; (**C**) Leave-one-out cross-validation of the PLS-DA model. The red star indicates the selected five-component PLS-DA model; (**D**) Model validation based on 100 permutation tests.

To further characterize overall differences in the chemical composition of digests prepared from different administration forms, untargeted metabolomics analysis was performed on samples collected at each digestion stage. The PCA score plot showed a separation tendency between the CSUP and CSW groups, indicating distinct metabolite profiles after in vitro digestion. The PLS-DA model further showed supervised discrimination among the groups. Leave-one-out cross-validation and a 100-permutation test were used to evaluate model performance and potential overfitting. Although the validation results supported non-random group discrimination, the PLS-DA results were interpreted as exploratory because of the limited sample size (Figure 4).

**Figure 5:**
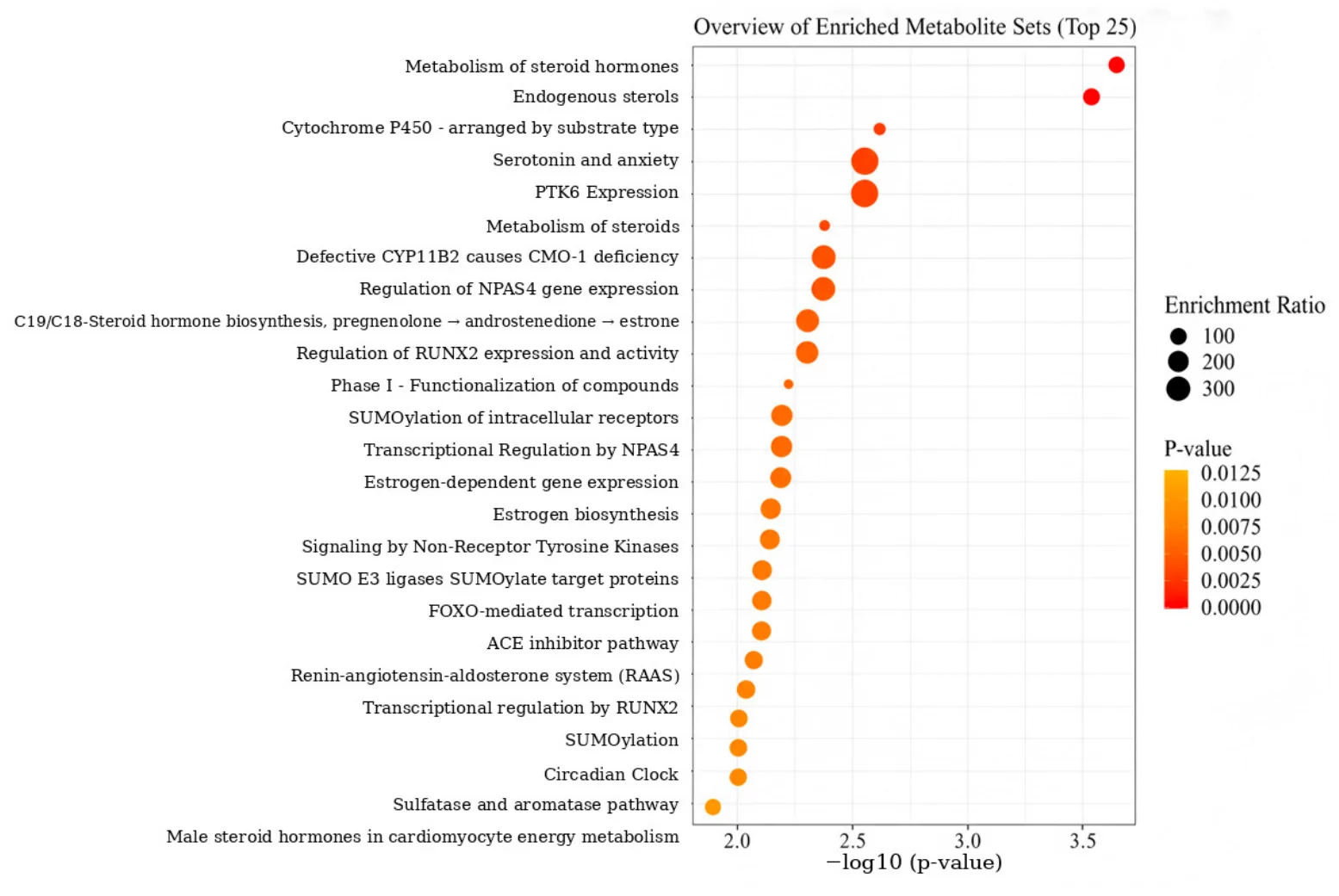
Enrichment analysis of differential metabolites using all detected and annotated metabolites in the untargeted metabolomics dataset as the background set. HMDB and KEGG annotations were used for metabolite and pathway mapping.

Enrichment analysis revealed that differential metabolites between CSUP and CSW digests were mainly enriched in metabolite sets related to steroid hormone metabolism, endogenous sterols, cytochrome P450-related substrate metabolism, steroid metabolism, and estrogen biosynthesis and regulation (Figure 5). Among these terms, the pathways “Metabolism of steroid hormones” and “Endogenous sterols” displayed high −log10 (*p*-value) values with red-shaded bubbles, indicating significant enrichment. Larger bubbles were observed for entries such as “Serotonin and anxiety”, “PTK6 Expression”, and “Metabolism of steroids”, indicating a high proportion of relevant differential metabolites enriched in these sets.

These results indicate marked differences in the metabolite profiles generated after in vitro digestion of CSUP and CSW. The differential compounds were mainly associated with sterols, steroids, and several lipid-related metabolites. This finding is consistent with the lipid class variations described above, suggesting that different administration forms may affect the release, transformation, and compositional distribution of lipid-related components from Coicis Semen during in vitro digestion. It should be noted that these enrichment results only reflect the functional annotation and statistical enrichment patterns of differential metabolites; their specific biological implications require further verification through targeted quantification, structural identification, and in vivo absorption studies.

### 3.4. Comparison of Antioxidant Activities of Samples at Different Digestion Stages

**Figure 6:**
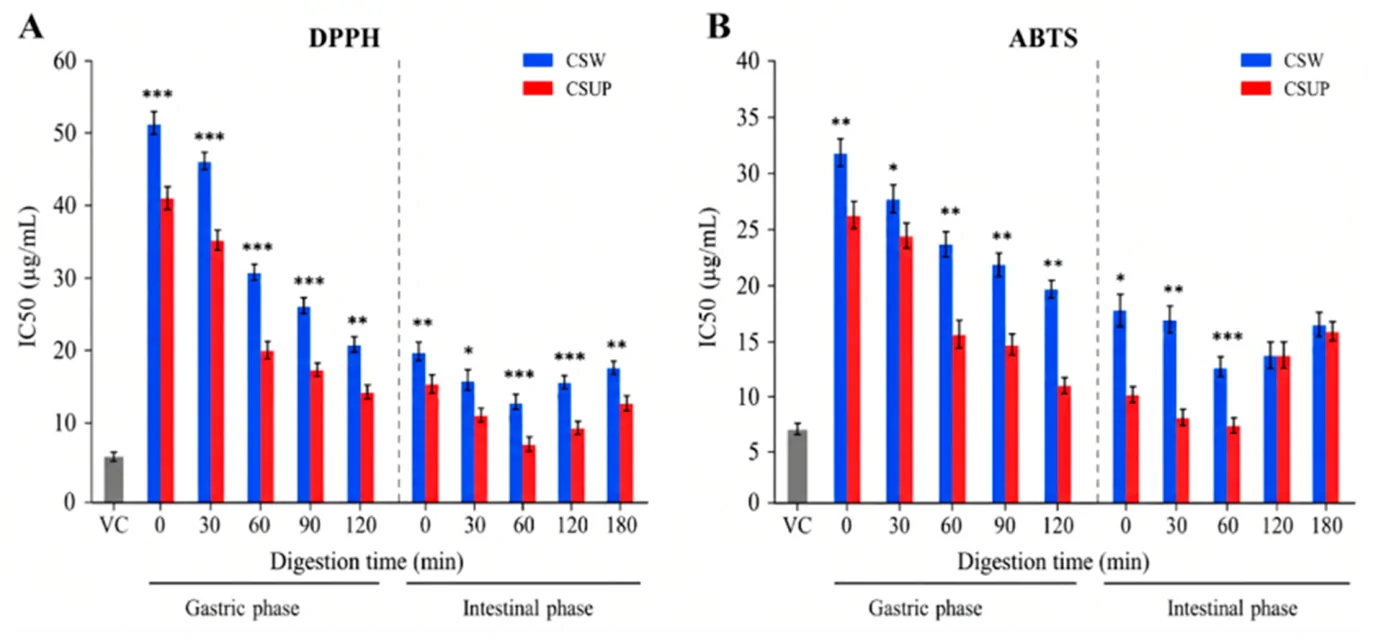
Antioxidant activity of samples at different digestion stages, expressed as IC_50_ values. (**A**) DPPH radical-scavenging assay; (**B**) ABTS radical-scavenging assay. Blue bars represent CSW, red bars represent CSUP, and gray bars represent vitamin C (VC), used as the positive control. Data are presented as mean ± SD (*n* = 3). Asterisks indicate statistically significant differences between CSW and CSUP at the same digestion time point (* *p* < 0.05, ** *p* < 0.01, *** *p* < 0.001). Lower IC_50_ values indicate stronger radical-scavenging activity.

DPPH and ABTS radical scavenging assays were used to assess the in vitro antioxidant activity of samples collected at different digestion stages. Radical scavenging capacity was quantified using IC_50_ values, with lower IC_50_ values indicating stronger antioxidant activity (Figure 6). The results showed that the DPPH and ABTS IC_50_ values of the CSUP group generally decreased as gastric digestion proceeded, reflecting a progressive improvement in radical scavenging capacity. The CSW group showed a comparable declining trend; however, its IC_50_ values were higher than those of the CSUP group at most sampling points. After transition to the intestinal digestion phase, the DPPH and ABTS IC_50_ values of the CSUP group decreased further, reached minimum values at the mid-intestinal stage, and then increased slightly, indicating stage-specific fluctuations in antioxidant activity during intestinal digestion.

Compared with CSW, CSUP exhibited lower IC_50_ values at most corresponding digestion time points, indicating that CSUP digests possessed superior in vitro radical scavenging capacity. This trend was largely consistent with the aforementioned results for measured peptide-related content and transformation of lipid-related constituents, suggesting that the improved antioxidant activity of CSUP may be attributable to increased measured peptide-related content, redistribution of lipid components, and co-release of other bioactive compounds. Nevertheless, the present findings cannot confirm the direct contribution of any single component to antioxidant activity. Further research integrating correlation analysis, quantification of key constituents, and activity verification is required to clarify the underlying mechanisms.

### 3.5. Chemical Equivalence, Bioequivalence, and Comprehensive Index Evaluation

**Figure 7:**
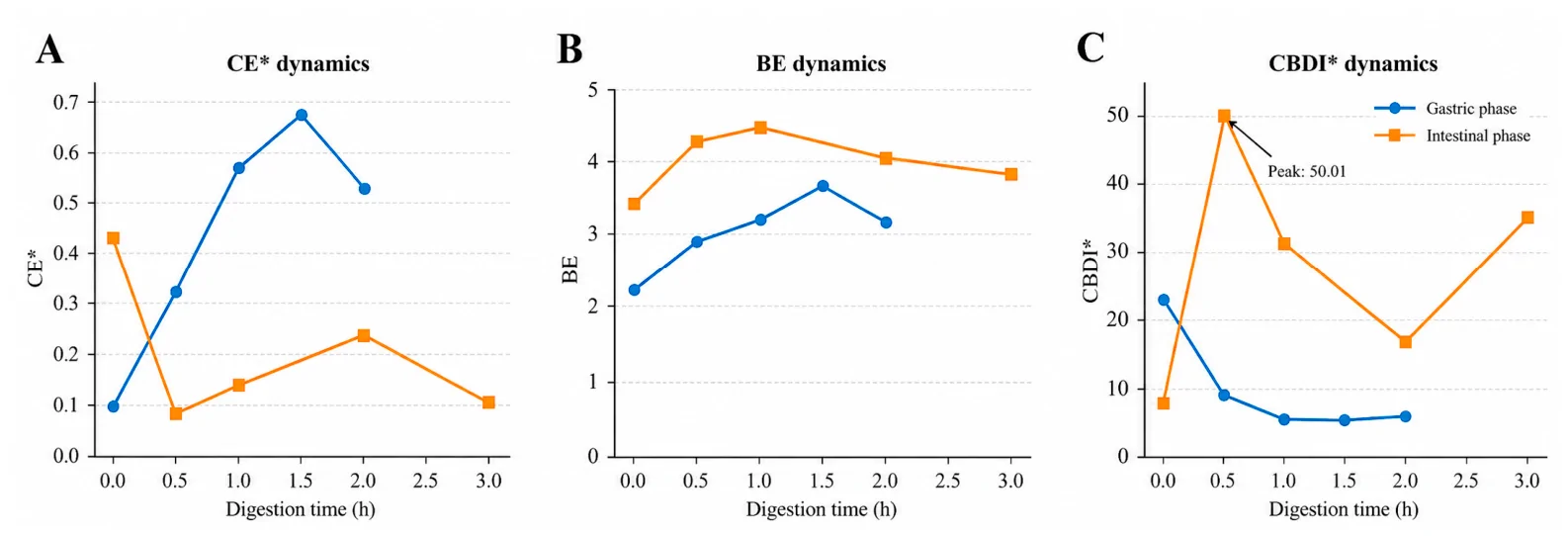
Dynamic changes in CE*, BE, and CBDI* during in vitro digestion of CSUP and CSW. (**A**) Dynamic changes in CE*; (**B**) Dynamic changes in BE; (**C**) Dynamic changes in CBDI*.

The Chemical Equivalence Index (CE*), Bioequivalence Index (BE), and Chemical–Biological Deviation Index (CBDI*) were calculated based on measured peptide-related content, lipid alterations, untargeted metabolomics, and antioxidant activity data. CE* increased progressively during gastric digestion, peaked at 1.5 h, and then declined slightly. During intestinal digestion, CE* was high at 0 h, decreased rapidly, and remained at a low level, suggesting that the disparity in chemical composition between CSUP and CSW became more pronounced upon entry into the intestinal phase (Figure 7A).

BE results indicated that CSUP exhibited higher composite in vitro radical-scavenging activity than CSW during both gastric and intestinal digestion. BE gradually increased during gastric digestion and remained higher during intestinal digestion than during gastric digestion, peaking at 1 h (Figure 7B). These results indicate stronger in vitro antioxidant capacity.

The CBDI* results showed that CBDI* values decreased rapidly and remained low during gastric digestion. During intestinal digestion, the index peaked at 0.5 h with a value of 50.01, then declined and increased again at 3 h (Figure 7C). These changes indicate a stage-dependent variation in the relationship between chemical similarity and composite in vitro radical-scavenging activity during digestion. Detailed calculation results of CE*, BE, and CBDI* for each group are shown in Table A4.

**Figure 8:**
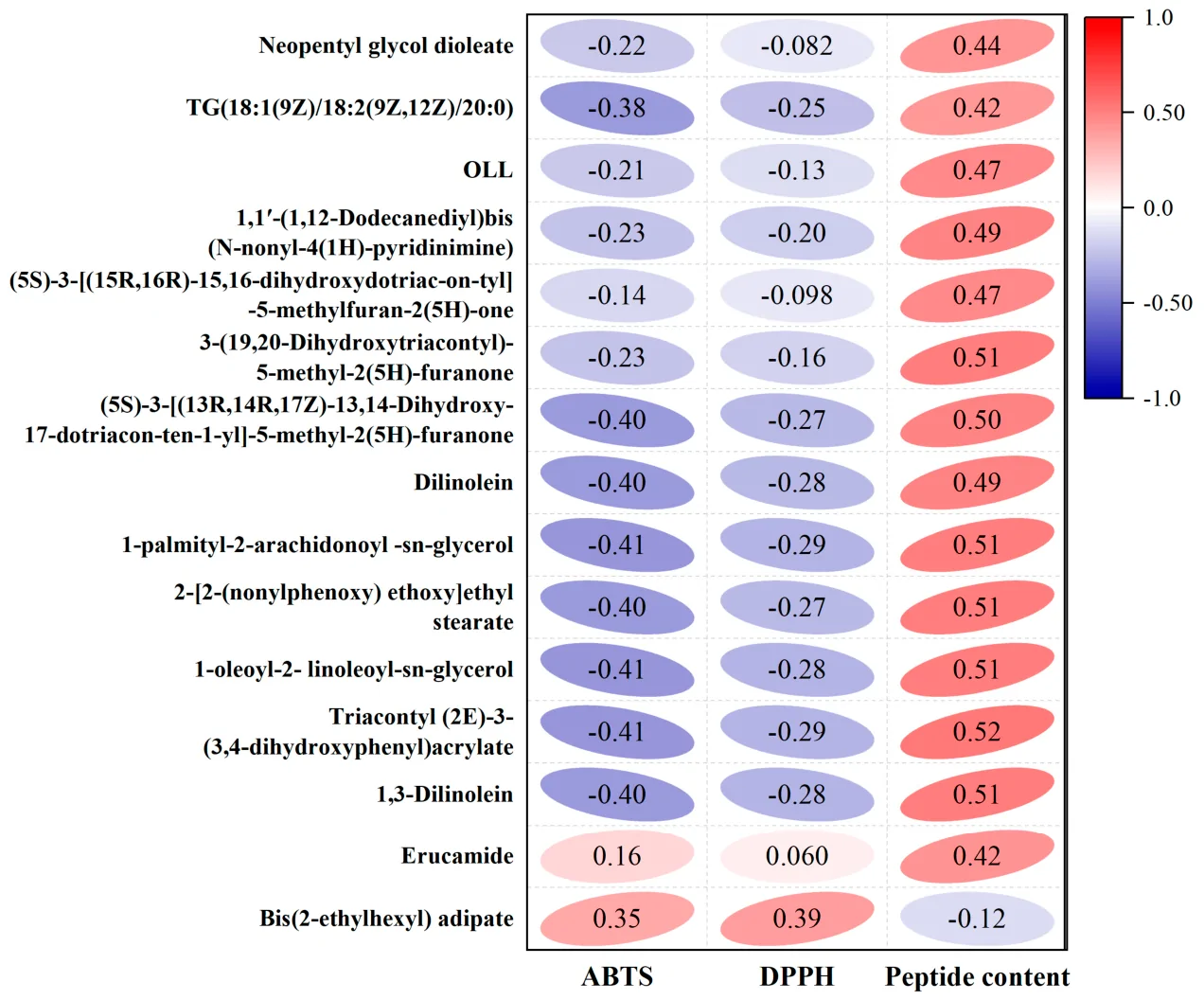
Correlation analysis between differential metabolites, antioxidant indicators and peptide content using pooled observations from CSUP and CSW. Red indicates a positive correlation, while blue indicates a negative correlation; the shade of color indicates the strength of the correlation. ABTS and DPPH are expressed as IC_50_ values; a lower IC_50_ value indicates stronger antioxidant activity.

Correlation analysis revealed that most differential compounds were positively correlated with peptide content and negatively correlated with the IC_50_ values of the ABTS and DPPH assays (Figure 8). Among them, Compounds 3, 4, 5, 6, 8, 10, and 11 showed strong correlations with peptide content and negative correlations with ABTS and DPPH IC_50_ values, indicating associations between these constituents and the antioxidant-related indicators. In contrast, Compound 1 showed positive correlations with ABTS and DPPH IC_50_ values and a weak negative correlation with peptide content, displaying a variation trend distinct from that of most differential metabolites.

## 4. Discussion

This study used the standardized INFOGEST 2.0 in vitro gastrointestinal digestion model to compare measured peptide-related content, lipid variation, metabolite profile differences, and in vitro antioxidant activity between CSW and CSUP during digestion. The results showed that CSUP exhibited higher measured peptide-related content, more pronounced redistribution of lipid classes, and stronger DPPH and ABTS radical scavenging capacities during gastric and intestinal digestion. These findings demonstrate differences between the two preparation forms in measured peptide-related content, lipid-related signals, metabolite profiles, and in vitro radical-scavenging activity under the tested conditions. The existing literature has shown that food matrix structure and processing treatments are key determinants of nutrient bioaccessibility [18,19].

The higher measured peptide-related content in CSUP reflects greater peptide-associated signals detected under the tested digestion conditions. Proteins in plant seeds typically form complex structures with starch, lipids, and cell wall components, which can limit contact between digestive enzymes and their substrates [20]. Previous studies have suggested that ultrafine pulverization may influence particle surface characteristics, wettability, and dispersibility, which could affect the interaction between food matrices and digestive fluids [21,22]. Therefore, the observed difference may result from the combined effects of matrix state, initial substrate availability, and processing history between the two preparations.

Lipidomic analysis revealed marked alterations in lipid classes in both groups at all digestion stages. During intestinal digestion, the relative TAG signal proportion in CSUP increased, while the relative distributions of other lipid-related components also changed across digestion stages. These findings indicate substantial stage-dependent redistribution of the detected lipid-related MS signals during the intestinal phase. Previous studies have indicated that lipid digestion efficiency is governed not only by lipases and bile salts but also by the distribution pattern and interfacial properties of lipids within the food matrix [23,24]. Variations in particle structure may be associated with differences in matrix structure and lipid distribution caused by different preparation processes. Accordingly, the different lipid-class patterns observed between CSUP and CSW may reflect their distinct matrix states and preparation processes, including the effects of decoction on the composition of CSW.

Untargeted metabolomics further revealed compositional disparities between the digests of the two groups. PCA and exploratory PLS-DA analyses indicated group-related separation patterns between CSUP and CSW, and enrichment analysis primarily implicated metabolite sets associated with steroid metabolism, endogenous sterols, and cytochrome P450. Previous studies have shown that sterols and fat-soluble bioactive compounds in plant-based foods are influenced by matrix structure, lipid release, and micelle formation during digestion, resulting in variable bioavailability profiles [25,26,27]. Therefore, the metabolite profile differences observed in the present study may be related to different degrees of release and transformation of lipophilic components between the two administration forms.

Antioxidant activity assays showed that CSUP exhibited lower IC_50_ values in the DPPH and ABTS assays at most digestion stages, indicating superior radical scavenging capacity. Previous research has demonstrated that gastrointestinal digestion of food proteins liberates short peptides with antioxidant activity, which exert antioxidant effects through hydrogen donation, electron transfer, and metal ion chelation [28,29]. In addition, plant-derived phenols, sterols, and some lipid metabolites are recognized to possess antioxidant potential [30,31]. Correlation analysis showed that most differential metabolites were positively correlated with peptide content and negatively correlated with DPPH and ABTS IC_50_ values, suggesting that these components were associated with antioxidant-related indicators.

In recent years, comprehensive evaluation strategies combining chemical fingerprinting, chemometrics, and bioactivity assessment have been widely applied in quality control research on traditional Chinese medicines and natural products. These methods emphasize correlation analysis between multicomponent chemical information and functional effects, thereby overcoming the limitation that single-component evaluation cannot adequately reflect the overall quality of complex systems [32,33,34]. The CE*, BE, and CBDI* indices were introduced in the present study as exploratory tools to summarize different aspects of the comparison between CSUP and CSW. CE* reflects their relative chemical similarity, with values closer to 1 indicating greater similarity. BE summarizes the relative in vitro radical-scavenging activity based on DPPH and ABTS assays, with values greater than 1 indicating higher activity in CSUP relative to CSW. CBDI* represents the ratio between antioxidant activity and chemical similarity and is used only to describe their stage-dependent relationship during digestion. A high CBDI* may result from lower chemical similarity, higher antioxidant activity, or both, and should not be interpreted as evidence of causal chemical–biological decoupling or a specific biological mechanism. These indices are study-specific exploratory metrics and are not established equivalence criteria.

It should be noted that CSUP and CSW differed in both processing method and matrix state. Equal starting-material equivalents did not ensure equal protein, lipid, or total-solid inputs into digestion. Because the baseline composition of the two preparations was not quantitatively standardized, the observed differences may reflect the combined effects of decoction, matrix structure, and initial substrate availability. Accordingly, the present findings should be interpreted as differences between the two preparation forms under the tested conditions rather than as evidence of an independent effect of ultrafine grinding.

## 5. Conclusions

Based on the standardized INFOGEST 2.0 in vitro gastrointestinal digestion model, this study compared the digestive behavior and in vitro antioxidant activities of Coicis Semen ultrafine powder suspension (CSUP) and water decoction (CSW). The results showed that CSUP had higher measured peptide content during digestion than CSW. Lipidomic analysis revealed dynamic stage-dependent changes in the relative MS signal distributions of TAGs, DAGs, and other lipid-related components in both sample groups, with pronounced changes occurring during intestinal digestion.

Untargeted metabolomics identified distinct metabolite profiles between CSUP and CSW digests, with differential metabolites primarily enriched in pathways related to steroid metabolism, endogenous sterols, and cytochrome P450. Antioxidant activity assays showed that CSUP yielded lower IC_50_ values in DPPH and ABTS assays at most digestion stages, corresponding to superior in vitro radical scavenging capacity. Correlation analysis using the pooled CSUP and CSW dataset revealed exploratory associations among several differential metabolites, peptide content, and DPPH/ABTS IC_50_ values.

The study-specific CE*, BE, and CBDI* indices provided descriptive information on chemical similarity, composite in vitro radical-scavenging activity, and their stage-dependent relationship, respectively. Collectively, CSUP and CSW exhibited marked differences in digestive behavior and antioxidant-related properties. CSUP showed lower DPPH and ABTS IC_50_ values than CSW at most digestion stages, indicating greater in vitro radical-scavenging activity under the tested conditions. This study provides comparative information for understanding differences between ultrafine powder suspension and water decoction of Coicis Semen during digestion.

## Figures and Tables

**Table 1:** Identification results of lipid-related components detected by UPLC-MS/MS.

NO.	Name	RT [min]	Ionization Mode	Formula	Calc. MW	Meas. MW	ppm	MS^2^
1	Bis(2-ethylhexyl) adipate	1.71	[M + H]^+^	C_22_H_42_O_4_	370.30894	371.31628	1.373	147.06581, 129.05502, 111.04436, 101.06003, 83.04942, 71.08572, 57.07006
2	Erucamide	1.74	[M + H]^+^	C_22_H_43_NO	337.33495	338.34268	1.43	321.31787, 303.30655, 205.19675, 149.13304, 121.10166
3	1,3-Dilinolein	1.94	[M + H]^+^	C_39_H_68_O_5_	616.50650	617.51373	−0.28	599.50281, 543.47711, 525.46631, 387.36374, 339.28955, 261.22189, 95.08549
4	Triacontyl (2E)-3-(3,4-dihydroxyphenyl)acrylate	2.25	[M + H]^+^	C_39_H_68_O_4_	600.51148	601.51874	−0.47	545.49396, 527.48315, 405.91040, 121.10101, 109.10108, 95.08552, 81.06989
5	1-oleoyl-2-linoleoyl-sn-glycerol	2.28	[M + H]^+^	C_39_H_70_O_5_	618.52203	619.52948	−0.48	601.51990, 568.79010, 545.49377, 527.48224, 415.39365, 339.28973, 135.11682, 121.10103, 109.10101, 95.08550, 81.06990
6	2-[2-(nonylphenoxy)ethoxy]ethyl stearate	2.31	[M + H]^+^	C_37_H_66_O_4_	574.49585	575.50323	−0.46	373.81793, 313.27411, 263.23727, 149.13254, 135.11685, 109.10110, 95.08549, 81.06987
7	1,3-Bis[(9Z,12Z)-9,12-octadecadienoyloxy]-2-propanyl (6Z,9Z,12Z)-6,9,12-octadecatrienoate	2.68	[M + H]^+^	C_57_H_96_O_6_	876.72020	877.72778	−0.56	689.48523, 642.75305, 597.48743, 541.46228, 502.23157, 398.19620, 269.01489
8	1-palmityl-2-arachidonoyl-sn-glycerol	2.69	[M + H]^+^	C_39_H_70_O_4_	602.52714	603.53467	−0.44	582.07184, 346.60443, 317.82104, 265.25241
9	Dilinolein	2.75	[M + H]^+^	C_39_H_72_O_5_	620.53769	621.54474	−0.45	562.52576, 413.57529, 388.10498, 339.28946, 95.08562
10	(5S)-3-[(13R,14R,17Z)-13,14-Dihydroxy-17-dotriaconten-1-yl]-5-methyl-2(5H)-furanone	2.76	[M + H]^+^	C_37_H_68_O_4_	576.51146	577.51855	−0.53	383.95950, 304.26855, 265.25302, 135.11673, 121.10111, 109.10114, 95.08553
11	3-(19,20-Dihydroxytriacontyl)-5-methyl-2(5H)-furanone	2.86	[M + H]^+^	C_35_H_66_O_4_	550.49583	551.50311	−0.51	383.53055, 274.83063, 239.23735, 137.13254, 109.10119, 95.08556
12	Stigmasta-3,5-diene	3.22	[M + H]^+^	C_29_H_48_	396.37542	397.38281	−0.46	315.30515, 287.27460, 257.22665, 229.19521, 215.17949, 189.16406, 175.14827, 161.13257, 147.11694, 135.11682, 121.10123, 109.10114, 95.08554
13	1,3-Bis(palmitoyloxy)-2-propanyl (5Z,8Z,11Z,14Z,17Z)-5,8,11,14,17-icosapentaenoate	3.31	[M + H]^+^	C_55_H_96_O_6_	852.72051	853.72723	−0.22	597.48816, 541.46234, 523.45123, 313.27344
14	(5S)-3-[(15R,16R)-15,16-dihydroxydotriacontyl]-5-methylfuran-2(5H)-one	3.46	[M + H]^+^	C_37_H_70_O_4_	578.52710	579.53448	−0.53	469.95313, 396.49115, 322.00363, 294.69272, 239.23775, 137.13245, 123.11683, 109.10111, 95.08550
15	(3β,22E)-Stigmasta-5,22-dien-3-yl 6-O-docosanoyl-β-D-glucopyranoside	3.78	[M + H]^+^	C_57_H_100_O_7_	896.74660	897.75391	−0.34	615.49957, 601.51825, 464.69614, 337.27502, 279.23221, 135.11697, 109.10113
16	1,3-Bis[(9Z,12Z)-9,12-octadecadienoyloxy]-2-propanyl (6Z,9Z,12Z)-6,9,12-octadecatrienoate	3.93	[M + H]^+^	C_57_H_96_O_6_	876.72038	877.72736	−0.35	859.88995, 684.85504, 597.48901, 541.46326, 523.45074
17	TG(16:1(9Z)/16:1(9Z)/18:1(9Z))	4.12	[M + H]^+^	C_53_H_96_O_6_	828.7206	829.72791	−0.11	573.49066, 551.51025, 261.22150
18	1,1′-(1,12-Dodecanediyl)bis(N-nonyl-4(1H)-pyridinimine)	4.23	[M + H]^+^	C_39_H_74_O_4_	606.55861	607.56586	−0.17	495.26297, 411.46878, 383.13864, 327.07825, 283.03114, 167.05518, 123.11678, 109.10114, 95.08551
19	(3β)-Stigmast-5-en-3-yl 6-O-docosanoyl-β-D-glucopyranoside	4.46	[M + H]^+^	C_57_H_102_O_7_	898.76215	899.76935	−0.45	675.83447, 617.51428, 601.51794, 405.61243, 337.27405
20	(3β)-Stigmast-5-en-3-yl 6-O-docosanoyl-β-D-glucopyranoside	4.64	[M + H]^+^	C_57_H_102_O_7_	898.76171	899.76941	−0.95	617.51312, 603.53638, 555.30066, 339.28958
21	Linolein	4.75	[M + H]^+^	C_57_H_98_O_6_	878.73547	879.74207	−0.99	599.50391, 543.47772, 399.36197, 337.27441
22	1,3-Bis(palmitoyloxy)-2-propanyl (5Z,8Z,11Z,14Z,17Z)-5,8,11,14,17-icosapentaenoate	5.06	[M + H]^+^	C_55_H_96_O_6_	852.71995	853.72729	−0.87	741.63458, 597.48895, 541.46301, 523.45123, 313.27371, 261.22156
23	3-(Heptadecanoyloxy)-2-[(9Z,12Z)-9,12-octadecadienoyloxy]propyl (9Z,12Z,15Z)-9,12,15-octadecatrienoate	5.37	[M + H]^+^	C_56_H_98_O_6_	866.73612	867.7431	−0.26	818.28070, 647.40289, 600.50372, 535.50024
24	unknown	5.53	[M + H]^+^	C_57_H_104_O_7_	900.77786	901.78485	−0.39	801.63208, 619.53375, 601.52051, 445.56323, 339.28952
25	unknown	5.7	[M + H]^+^	C_55_H_102_O_7_	874.76215	875.76984	−0.46	788.85797, 613.68024, 577.52209, 339.28989, 269.01450
26	OLL	5.95	[M + H]^+^	C_57_H_100_O_6_	880.75081	881.75806	−1.34	599.50354, 543.47668, 525.46552, 387.36252, 339.28928
27	PLL	6.2	[M + H]^+^	C_55_H_98_O_6_	854.73516	855.74261	−1.38	599.50256, 543.47778, 525.46704, 313.27386, 261.22122
28	TG(16:1 (9Z)/16:1 (9Z)/18:1 (9Z))	6.27	[M + H]^+^	C_53_H_96_O_6_	828.72051	829.72754	−0.22	573.49066, 551.51025, 388.32684, 261.22150
29	1-[(9Z)-heptadecenoyl]-2-oleoyl-3-linoleoyl-sn-glycerol	6.88	[M + H]^+^	C_56_H_100_O_6_	868.75179	869.75897	−0.23	742.70789, 599.50159, 403.20728, 327.28983, 121.10128
30	Triglyceride OLO,sn	7.48	[M + H]^+^	C_57_H_102_O_6_	882.76670	883.77399	−1.06	865.76337, 613.67572, 601.51886, 545.49402, 527.48077, 339.28949
31	1-Palmitoyl-2-Oleoyl-3-Linolein	7.78	[M + H]^+^	C_55_H_100_O_6_	856.75123	857.75848	−0.89	601.51746, 545.49329, 527.48151, 509.47217, 313.27313
32	TG(18:1 (9Z)/14:0/18:1 (9Z))	8.13	[M + H]^+^	C_53_H_98_O_6_	830.73622	831.74316	−0.15	575.50281, 551.50305, 501.46710, 313.27371, 263.23740
33	unknown	8.71	[M + H]^+^	C_56_H_102_O_6_	870.76728	871.77429	−0.41	613.66608, 520.21729, 422.78873, 109.10118
34	Glycerinetrioleate	9.48	[M + H]^+^	C_57_H_104_O_6_	884.78242	885.78943	−0.99	603.53656, 529.49994, 339.28979, 265.25290, 247.24207, 149.13266, 135.11690, 121.10116
35	TG(18:2 (9Z,12Z)/18:2 (9Z,12Z)/20:0)	9.72	[M + H]^+^	C_59_H_106_O_6_	910.79846	911.8064	−0.52	599.50452, 543.47803, 525.46741, 369.33688, 261.22156, 149.13255, 135.11690, 121.10126, 109.10125
36	3-Palmito-1,2-Diolein	9.89	[M + H]^+^	C_55_H_102_O_6_	858.76685	859.77423	−0.92	603.53680, 577.51831, 529.49768, 339.29001, 265.25259
37	2,3-Dipalmito-1-olein	10.33	[M + H]^+^	C_53_H_100_O_6_	832.75166	833.7594	−0.39	809.66351, 705.03912, 577.51959, 551.50385, 313.27438, 109.10113
38	TG(18:1 (9Z)/18:2 (9Z,12Z)/20:0)	11.8	[M + H]^+^	C_59_H_108_O_6_	912.8145	913.82147	−0.1	631.56207, 604.11200, 530.24591, 135.11713, 121.10129
39	TG(18:1 (9Z)/18:2 (9Z,12Z)/20:0)	12.39	[M + H]^+^	C_59_H_108_O_6_	912.8141	913.82135	−0.54	808.25610, 601.52203, 545.49365, 527.48431, 509.47339, 369.33670, 109.10113
40	1,2-Dioleoyl-3-stearoyl-rac-glycerol	12.48	[M + H]^+^	C_57_H_106_O_6_	886.79831	887.80573	−0.71	605.55109, 529.49939, 482.54630, 341.30533, 265.25244, 135.11696, 121.10120, 109.10111
41	1-Palmitoyl-2-oleoyl-3-stearoylglycerol	13.05	[M + H]^+^	C_55_H_104_O_6_	860.78275	861.79034	−0.63	605.55133, 577.51721, 330.02704, 109.10115
42	Stigmasta-3,5-diene	13.64	[M + H]^+^	C_29_H_48_	396.37544	397.38257	−0.4	257.22702, 215.17958, 201.16451, 175.14841, 161.13260, 147.11685, 135.11688, 121.10110, 109.10117
43	Neopentyl glycol dioleate	15.86	[M + H]^+^	C_41_H_76_O_4_	632.57423	633.58154	−0.21	586.09186, 546.32147, 391.30981, 293.87903, 149.13228, 121.10143, 109.10105, 95.08550

## Data Availability

The data presented in this study are available on request from the corresponding author due to university policy.

## Abbreviations

The following abbreviations are used in this manuscript:

CSUP	Coicis Semen ultrafine powder suspension
CSW	Coicis Semen water decoction
DPPH	2,2-diphenyl-1-picrylhydrazyl
ABTS	2,2′-azino-bis (3-ethylbenzothiazoline-6-sulfonic acid)
UPLC-MS/MS	Ultra-performance liquid chromatography–tandem mass spectrometry
HMDB	Human Metabolome Database
PCA	Principal component analysis
PLS-DA	Partial least squares discriminant analysis
CE*	Chemical Equivalence Index
BE	Bioequivalence Index
CBDI*	Chemical-Biological Deviation Index
TAGs	Triacylglycerols
DAGs	Diacylglycerols
MAGs	Monoacylglycerols
FFAs	Free fatty acids
SIF	Simulated intestinal fluid
SGF	Simulated gastric fluid
APCI	Atmospheric pressure chemical ionization
dd-MS2	Data-dependent MS/MS

## Appendix A

### Appendix A.1. Preparation of Simulated Gastric and Intestinal Fluids

**Table A1:** Preparation of stock solution for simulated gastric fluid (SGF).

Components	Concentration (mmol/L)	Molecular Weight	Solution Volume (mL)	Mass (g)	Volume in Reference Protocol (mL)	Volume Required for Preparing 1 L (mL)
KCl	6.9	74.5	840	0.4318	6.9	276
KH_2_PO_4_	0.9	136	120	0.0147	0.9	36
NaHCO_3_	25	84	1510	3.1710	12.5	500
NaCl	47.2	58.5	1510	4.1694	11.8	472
MgCl_2_·6H_2_O	0.1	203	60	0.0012	0.4	16

The SGF stock solution was prepared at 1.25-fold concentration and diluted with distilled water before use. The pH was adjusted to 3.0 with HCl.

**Table A2:** Preparation of stock solution for simulated intestinal fluid (SIF).

Components	Concentration (mmol/L)	Molecular Weight	Solution Volume (mL)	Mass (g)	Volume in Reference Protocol (mL)	Volume Required for Preparing 1 L (mL)
KCl	6.8	74.5	500	0.2533	6.8	136
KH_2_PO_4_	0.8	136	100	0.0109	0.8	16
NaHCO_3_	85	84	1000	7.14	85	850
NaCl	38.4	58.5	500	1.1232	38.4	192
MgCl_2_·6H_2_O	0.33	203	200	0.0134	0.33	22

The SIF stock solution was prepared at 1.25-fold concentration and diluted with distilled water before use. The pH was adjusted to 7.0 with NaOH.

### Appendix A.2. Comprehensive Integrated Evaluation of CE*, BE, and CBDI*

**Table A3:** Description of the Calculation Formulas for CE*, BE, and CBDI*.

Index	Formula	Meaning
CE*	CE* = 1 − Σ wi·|(CiS − CiR)/(CiS + CiR + ε)|	Chemical composition similarity between CSUP and CSW
BE	BE = Σ vj·(BjS/BjR)	Weighted biological effect ratio
CBDI*	CBDI* = BE/(CE* + ε)	Chemical–biological relationship index

Calculation notes: (1) CE* was calculated based on three chemical categories: peptide-related content, lipid-related signals, and metabolite profiles. These three categories were assigned equal weights, and equal weighting was applied to individual indicators within each category. (2) BE was calculated based on DPPH and ABTS radical-scavenging assays. Because both indicators were expressed as IC_50_ values, they were converted into activity-oriented values before integration. DPPH and ABTS were assigned equal weights (0.5 each). (3) CBDI* was used as a descriptive index reflecting the numerical relationship between chemical similarity and composite radical-scavenging activity.

**Table A4:** Calculated values of CE*, BE, and CBDI*.

Phase	Time (h)	CE*	BE	CBDI*
Gastric	0	0.0971	2.2291	22.9465
Gastric	0.5	0.3218	2.9059	9.0288
Gastric	1	0.5726	3.1932	5.5770
Gastric	1.5	0.6788	3.6497	5.3765
Gastric	2	0.5307	3.1513	5.9376
Intestinal	0	0.4299	3.3924	7.8904
Intestinal	0.5	0.0854	4.2691	50.0128
Intestinal	1	0.1419	4.4322	31.2438
Intestinal	2	0.2389	4.0290	16.8669
Intestinal	3	0.1086	3.8141	35.1319
